# The Impact of Long COVID on Cognitive Performance and Sleep Quality: An Analysis of the Rancagua Chilean Study (RACHIS)

**DOI:** 10.7759/cureus.55089

**Published:** 2024-02-27

**Authors:** Héctor Aceituno, Andrea Barrancas, Fernando Quiroz-Bravo, Dairene Rigaud, Denis Pérez-Cuesta, Aline Tobar-Bustamante, Marcela Osores-Espinoza, Carla Figueroa-Torres, Catalina Rojas-Catejo, Jorge Cisneros-Zamora

**Affiliations:** 1 Neurological Surgery, Hospital San Juan de Dios, Curico, CHL; 2 Neurology, Hospital Regional Libertador Bernardo O'Higgins, Rancagua, CHL; 3 General Medicine, Centro de Salud Familiar N°1 (CESFAM N°1), Rancagua, CHL; 4 Surgery, Hospital Regional Libertador Bernardo O'Higgins, Rancagua, CHL; 5 Medicine, Universidad del Alba, Santiago, CHL

**Keywords:** covid-19, mild symptoms, outpatient patients, sleep disorder, cognitive deficit, long covid

## Abstract

Background

Infection with the severe acute respiratory syndrome coronavirus 2 (SARS-CoV-2) can lead to prolonged symptoms post-recovery, commonly known as long-term coronavirus disease 2019 (COVID-19) or “long COVID.” Neuropsychiatric consequences of long COVID include cognitive dysfunction and sleep disturbances, which significantly impair daily living. This study aimed to explore the impact of long COVID on cognitive performance and sleep quality in patients receiving outpatient care.

Material and methods

This study involved a random sample of 138 of 363 patients, corresponding to 38% of the cohort, who tested positive for SARS-CoV-2 via polymerase chain reaction (PCR) between May 2020 and April 2021. These unvaccinated, non-hospitalized individuals, predominantly exhibiting mild disease symptoms, were prospectively assessed 11 months post-positive PCR test. After informed consent, demographic data, memory, and concentration impairment levels were collected through interviews. Participants reporting cognitive symptoms underwent the Mini-Mental State Examination (MMSE), the Montreal Cognitive Assessment (MOCA), and the Pittsburgh Sleep Quality Index. Statistical analyses were conducted, including Student’s t-test, Chi-square, Fisher’s test, Kruskal-Wallis test, and Pearson correlation coefficient, with a significance threshold set at p<0.05.

Results

Of the 138 participants, 76 (55.1%) were female and 62 (44.9%) were male. The mean age was 45.9 years (± 13.0), with an average educational attainment of 10.4 years (± 3.7). Roughly 50% of the patients reported significant memory and concentration issues (p<0.001). Thirty-three participants underwent detailed cognitive assessments, revealing a 2:1 female-to-male ratio and a significantly higher prevalence of depression in female participants. Cognitive deficits were diagnosed in five (15.2%) participants via the MMSE and in 26 (78.8%) via the MOCA test, with notable deficits in visuospatial/executive functions, language repeat, and deferred recall (p<0.001). A lower educational level was correlated with higher cognitive deficits (p=0.03).

Conclusion

The study findings reveal that cognitive impairments, as a consequence of COVID-19, can persist up to 11 months post-infection. The MOCA test proved more effective in diagnosing these deficits and requires adjustments based on educational background. Sleep parameters remained largely unaffected in this cohort, likely attributed to the mild nature of the initial symptoms and the outpatient management of the disease.

## Introduction

Long-term coronavirus disease 2019 (COVID-19), or “long COVID,” is a condition that arises in individuals with a history of probable or confirmed infection by the severe acute respiratory syndrome coronavirus 2 (SARS-CoV-2), the virus responsible for COVID-19 [1]. This condition manifests through the persistence of symptoms such as fatigue, dyspnea (difficulty breathing), cognitive impairment (commonly referred to as “brain fog”), post-exertional malaise, memory issues, musculoskeletal pain or spasms, cough, sleep disturbances, tachycardia or palpitations (rapid heart rate), altered smell or taste perception, headache, chest pain, and depression. Notably, individuals with long COVID face a heightened risk of developing neurological and psychiatric issues compared to those with other infectious respiratory diseases [2,3].

Long COVID typically emerges three months after the initial symptom onset, persists for at least two months, and cannot be attributed to other diagnoses [1]. Its prevalence varies based on geographic location, ranging from 9% to 63%, and can persist for up to two years in some cases. Factors such as the SARS-CoV-2 variant, particularly the Omicron variant and its associated viral load, along with age, gender, smoking habits, reactivation of the Epstein-Barr virus, obesity, diabetes, and hypertension, have influenced the fluctuations in COVID-19 prevalence during the pandemic’s three waves [2,4]. Studies estimate that unvaccinated individuals have a 41.8% risk of developing long COVID (95% CI: 37.0-46.7%), whereas vaccination prior to infection reduces this risk (risk reduction: 0.71) [4,5].

Severe cases of long COVID, particularly those requiring mechanical ventilation, are more prevalent among hospitalized individuals, followed by outpatient cases with moderate to mild symptoms [2]. Among patients with persistent symptoms, cognitive issues are reported in 9% to 50% of cases, and 11% to 13% experience sleep disorders. These conditions degrade the quality of life and significantly strain healthcare systems worldwide [6].

Although direct infection of the central nervous system by the SARS-CoV-2 virus remains unproven, one hypothesis suggests that invasion of the gastrointestinal epithelium, persisting for an average of 30.9 days, may disrupt the microbiome. This disruption could lead to dysregulation of the gut-brain axis, potentially causing persistent neurological symptoms. Additionally, the reactivation of herpesviruses, such as the Epstein-Barr virus and varicella-zoster virus, which remain latent in 90% of the population, might trigger neuroinflammation. This inflammation, associated with elevated antibodies, could be linked to memory issues and fatigue in long COVID, as epidemiological studies suggest [4].

The severity of initial COVID-19 symptoms can influence the prevalence and presentation of cognitive issues, as seen in patients with brain fog [7]. Clinical phenotypes featuring memory, attention, and executive function issues have been proposed [8]. Considering long COVID as a multisystemic disease, these cognitive disorders may play a role in the chronicity of symptoms. This situation highlights the potential for accelerated neurodegenerative mechanisms and the activation of previously underexplored genetic vulnerabilities following SARS-CoV-2 infection [8].

Sleep disturbances are associated with anxiety and depression post-hospital discharge [9]. The prevalence of these disturbances is impacted by the severity of initial COVID-19 symptoms, the need for oxygen support, oxygen saturation levels upon admission, and levels of C-reactive protein, serum ferritin, and D-dimer in the blood [9]. Identified risk factors for these patients include female gender, older age, lower education level, diabetes, obesity, and preexisting hypertension. These factors contribute to the varying frequency of sleep disorders among the populations studied [9]. This study aimed to explore the impact of long COVID-19 on cognitive performance and sleep quality in outpatient care recipients.

## Materials and methods

This study is part of the Rancagua Chilean Study (RACHIS) on persistent neurocognitive symptoms among COVID-19 patients, conducted at Family Health Center No. 1 (CESFAM No. 1) in Rancagua, Chile. The Scientific Ethics Committee of the Municipal Health Corporation of Rancagua (CORMUN), Chile, approved the study (approval no.: 23122020), adhering to the principles outlined in the Declaration of Helsinki [10].

**Figure 1:**
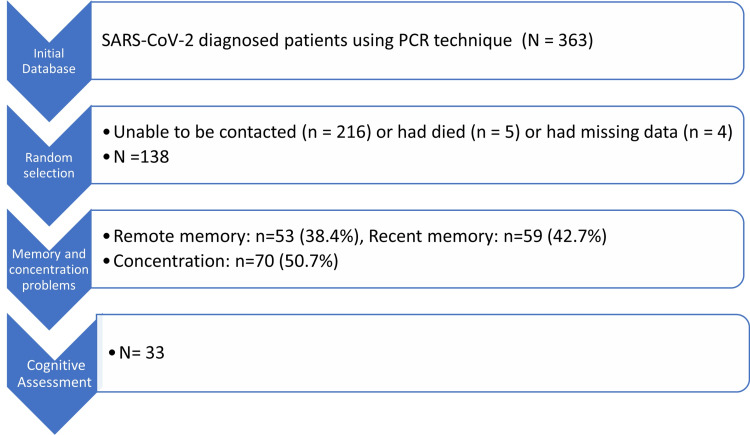
Sample selection flow diagram PCR: polymerase chain reaction; SARS-CoV-2: severe acute respiratory syndrome coronavirus 2

The study included a random sample of 138 unvaccinated, non-hospitalized patients with mild symptoms from an institutional database of 363 polymerase chain reaction (PCR)-confirmed COVID-19 cases recorded between May 2020 and April 2021. These patients underwent prospective neurocognitive evaluations 11 months post-PCR confirmation of the disease (Figure 1).

Exclusion criteria included individuals with mental disabilities (such as schizophrenia, bipolar disorder, dissociative disorders, compulsive disorder, psychosis, or paranoia), vascular encephalopathy or neurodegenerative diseases, and those under 18 years of age.

Study protocol

Prior to participation, all participants provided written informed consent. The researchers administered a general questionnaire developed by the study authors to gather demographic information and details about comorbidities through personal interviews. The questionnaire also inquired about memory and concentration issues, quantifying severity on a Likert scale from 1 (none) to 5 (excessive). Anxiety and depression were evaluated using two validated tools: the Goldberg questionnaire, defining anxiety with scores above four and depression with scores above three [11], and the Patient Health Questionnaire-9, which assesses depression severity on a scale ranging from none/minimal (0-4) to severe depression (20-27) [12].

A neurology specialist conducted cognitive assessments using the Mini-Mental State Examination (MMSE) in Spanish, with a total possible score of 30 points and a cutoff of 25 indicating cognitive deficit. The MMSE assesses temporal orientation, spatial orientation, retention, calculation/attention, memory recall, and language components [13]. The Montreal Cognitive Assessment (MOCA) was also administered, scoring from 0 to 30, with a cutoff for mild cognitive deficit at 26. An additional point was added to the total score for individuals with less than 12 years of education [14]. The MOCA evaluates visuospatial/executive functions, naming, attention numbers, attention letters, attention subtract, language repeat, language fluency, abstraction, deferred recall, and orientation [14].

Participants also completed the Pittsburgh Sleep Quality Index, which uses scores ranging from 0 to 21 to evaluate subjective sleep quality, latency, duration and efficiency of sleep, sleep disturbances, and daytime somnolence, with higher scores indicating greater sleep difficulties [15].

Statistical analysis

The data were collated in a structured database. The sample size was calculated using contingency table tests to achieve a statistical power of 95% and an alpha error of 0.05. Continuous variables were analyzed with the Student’s t-test, and dichotomous variables were assessed using the Chi-square test, applying Fisher’s adjustment when necessary. The Kruskal-Wallis test was used for Likert scale variables, and the Pearson correlation coefficient was employed to determine associations between numerical variables. All tests were two-tailed.

## Results

**Figure 2:**
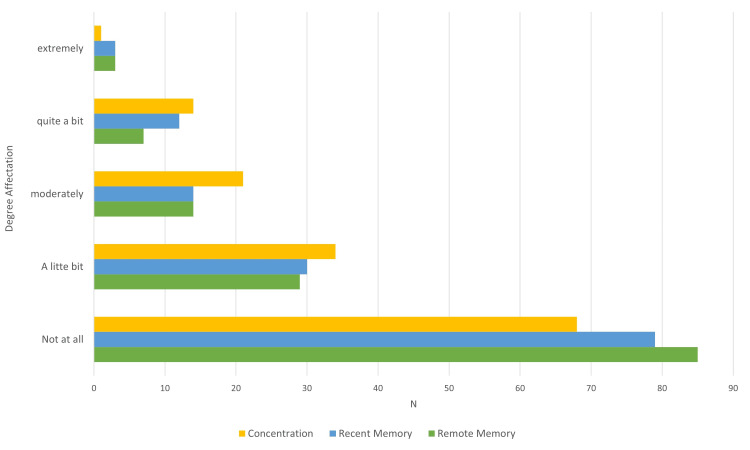
Degree of reported affectation in concentration, recent and remote memory The difference was significant when p≤0.05 when values in normal patients were compared with the degree of reported affectation

This study analyzed a total of 138 cases, consisting of 76 female (55.1%) and 62 male (44.9%) participants. The mean age of the patients was 45.9 years (standard deviation of ± 13.0 years). The mean educational attainment was 10.4 years (± 3.7 years). The most common comorbidities identified were hypertension (31.2%, 43 cases), diabetes (18.8%, 22 cases), and hypothyroidism (14.5%, 20 cases), with a statistically significant prevalence (p<0.001). Anxiety disorder was present in 86 cases (62.3%) and depression in 82 cases (59.4%), including 25 mild (18.1%), 30 moderate (21.7%), 17 moderate-severe (12.3%), and 10 severe cases (7.2%), showing a statistically significant occurrence (p<0.001). Nearly half of the patients reported issues with both recent and remote memory and concentration problems, with these issues presenting at significant levels of severity (Figure 2).

Cognitive assessments

A subgroup of 33 patients underwent comprehensive cognitive evaluations. This group had a female-to-male ratio of 2:1 and a significantly higher rate of depression among female patients, including six mild (18.2%), nine moderate (27.3%), five moderate-severe (15.2%), and four severe cases (12.1%; p<0.001) (Table 1).

**Table 1:** Demographic characteristics and comorbidities in patients with cognitive assessment Continuous variables are presented in mean ± standard deviation and range. Discrete variables are presented as numbers and percentages. The difference was significant when p≤0.05 SD: standard deviation

Category		Total, N=33	Male participants, n=11	Female participants, n=22	p-value
Mean age in years, ± SD		48.7 ± 11.9	49.1 ± 13.9	48.5 ± 11.4	0.581
Mean education in years, ± SD		11.2 ± 3.2	12.2 ± 3.3	10.6 ± 3.1	0.647
Employment, n (%)		18 (54.5)	9 (81.8)	9 (40.9)	0.300
Comorbidities	Anxiety, n (%)	23 (69.7)	6 (54.5)	17 (77.3)	0.174
	Depression, n (%)	18 (54.5)	3 (27.3)	15 (68.2)	0.031
	Hypertension, n (%)	12 (36.4)	4 (36.4)	8 (36.4)	0.653
	Obesity, n (%)	11 (33.3)	4 (36.4)	7 (31.8)	0.563
	Diabetes, n (%)	10 (30.3)	4 (36.4)	6 (27.3)	0.440
	Hypothyroidism	8 (24.2)	-	8 (36.4)	0.023
Psychobiological habits	Alcohol, n (%)	19 (57.6)	7 (63.6)	12 (54.5)	0.453
	Tabaco, n (%)	5 (15.2)	1 (9.1)	4 (18.2)	0.450
	Marijuana, n (%)	4 (12.1)	2 (18.2)	2 (9.1)	0.407

The MMSE identified cognitive deficits in five patients (15.2%), with significant discrepancies in total scores, notably in the memory recall domain (Table 2).

**Table 2:** Cognitive domains assessed by the MMSE in COVID-19 patients *Fisher's exact test. The difference was significant when p≤0.05 COVID-19: coronavirus disease 2019, N/A: not applicable, SD: standard deviation, MMSE: Mini-Mental State Examination

Cognitive domains	Range	Normal, n=28 (mean ± SD)	Cognitive impairment, n=5 (mean ± SD)	p-value
Temporal orientation	0-5	5.0 ± 0.6	4.0 ± 0.7	0.30
Spatial orientation*	0-5	5.0 ± 0.0	4.8 ± 0.5	0.152
Retention	0-3	3.0 ± 0.0	3.0 ± 0.0	N/A
Calculation/attention	0-5	4.8 ± 0.5	5.0 ± 0.0	0.591
Memory recall	0-3	2.5 ± 0.7	1.0 ± 1.2	<0.001
Language	0-9	8.0 ± 0.98	7.4 ± 1.1	0.341
Total score	0-30	28.1 ± 1.5	24.4 ± 0.6	<0.001

The MOCA test revealed cognitive deficits in 26 participants (78.8% of those with positive cases), with significant differences in total scores. The domains most affected by the SARS-CoV-2 infection included visuospatial/executive functions, language repeat, and deferred recall (Table 3).

**Table 3:** Cognitive domains assessed by the MOCA test in COVID-19 patients *Fisher's exact test. The difference was significant when p≤0.05 COVID-19: coronavirus disease 2019, SD: standard deviation, MOCA: Montreal Cognitive Assessment

Cognitive domains	Range	Normal, n=7 (mean ± SD)	Cognitive impairment, n=26 (mean ± SD)	p-value
Visuospatial/executive	0-5	4.7 ± 0.3	2.4 ± 1.2	<0.001
Naming	0-3	2.8 ± 0.4	2.6 ± 0.9	0.780
Attention numbers	0-2	1.7 ± 0.7	1.4 ± 0.3	0.184
Attention letters*	0-1	1.0 ± 0.0	0.9 ± 0.1	0.616
Attention subtract	0-3	2.7 ± 0.4	2.0 ± 0.9	0.379
Language repeat	0-2	1.4 ± 0.5	0.6 ± 0.7	0.031
Language fluency*	0-1	1.0 ± 0.0	0.7 ± 0.4	0.081
Abstraction	0-2	1.2 ± 0.7	1.3 ± 0.7	0.829
Deferred Recall	0-5	4.1 ± 0.7	1.8 ± 1.3	<0.001
Orientation	0-6	6.0 ± 0.0	5.7 ± 0.5	0.373
Total	0-30	27.4 ± 1.3	20.4 ± 3.3	0.003

**Figure 3:**
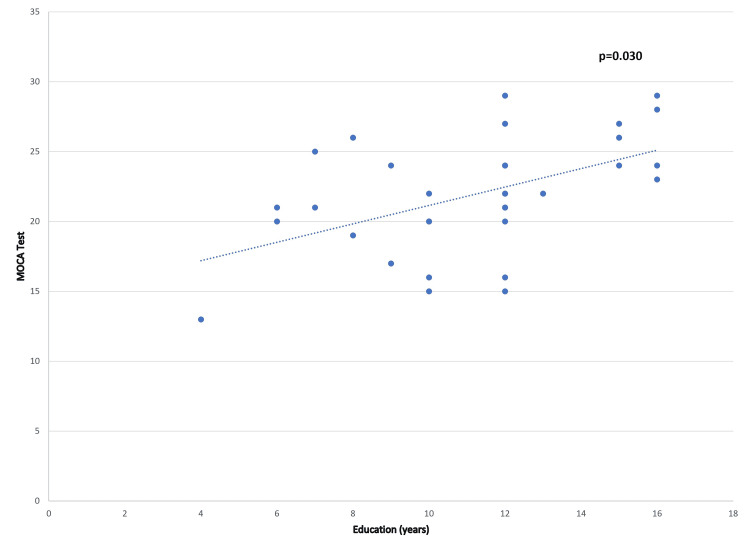
Relationship between years of education and scores on the MOCA test in long COVID Pearson correlation: The correlation between the MOCA test score and years of education was significant when the p-value was ≤0.05. long COVID: long-term coronavirus disease 2019, MOCA: Montreal Cognitive Assessment

A positive correlation was observed between years of education and the diagnosis of cognitive deficits based on the overall MOCA test scores (Figure 3).

However, no significant associations were found between cognitive deficits and other demographic factors or comorbidities, including depression and anxiety.

Sleep quality

Regarding sleep quality, eight patients (24.2%) reported insomnia related to sleep initiation, 10 patients (30.3%) had issues with sleep maintenance, and another 10 patients (30.3%) experienced mixed insomnia. Among those diagnosed with cognitive deficits via the MOCA test, lower scores were noted on the Pittsburgh Sleep Quality Index. No significant differences were observed in other variables among the patient groups (Table 4).

**Table 4:** Sleep quality parameters assessed in COVID-19 patients diagnosed with cognitive impairment through the MOCA test The difference was significant when p≤0.05 COVID-19: coronavirus disease 2019, SD: standard deviation, MOCA: Montreal Cognitive Assessment

Components	Normal (n=7)		Cognitive impairment (n=26)		p-value
	Mean ± SD	Range	Mean ± SD	Range	
Hours it takes to fall asleep	1.4 ± 0.93	0.5-3	1.9 ± 1.2	0.2-4	0.731
Hours slept	4.7 ± 0.75	4.0-6.0	4.3 ± 1.3	2.0-7.0	0.562
Sleep efficiency (%)	57.5 ± 14.5	38.0-77.5	61.1 ± 26.2	21-100	0.458
Pittsburgh total score	16.0 ± 2.3	14.0-19.0	15.8 ± 3.8	8.0-21.0	0.043

## Discussion

This study underscores the prevalence of cognitive deficits in three-quarters of the assessed population, correlating with their educational attainment at least 11 months post-COVID-19 infection. These patients, managed on an outpatient basis, demonstrated the persistence of symptoms while maintaining relatively good sleep quality.

In global assessments of patients with SARS-CoV-2, the MMSE and the MOCA are pivotal tools for evaluating varying degrees of cognitive deficits [16]. These assessments gauge functions such as attention, executive functions, processing speed, learning, and memory, playing a crucial role in the early detection of cognitive issues [16]. Our findings indicate that 11 months post-COVID-19 diagnosis, adult patients exhibit impairments in working memory, attention, and processing speed. The variability in individual responses results in different frequencies, severities, and affected cognitive aspects [16], delineating the impact on each population.

However, these assessments are influenced by factors including age, educational level, baseline cognitive state, comorbidities, symptom severity and duration, hospital management, the occurrence of delirium during hospitalization, and potential observer error, which may affect the comparability of results. Notably, anxiety and depression do not seem to influence these outcomes [16].

The MOCA test is particularly sensitive to mild cognitive deficits, whereas the MMSE is better suited for identifying severe deficits [16]. The MOCA test boasts a sensitivity of 92% (95% CI: 86-96) and a specificity of 90% (95% CI: 86-93), rendering it highly effective for population screening [17]. The influence of educational level on test outcomes is notable, serving as a protective factor. Individuals with higher educational attainment often display a cognitive reserve, scoring better on these assessments [17]. To address this bias, proposals suggest adjusting the total cutoff score based on educational level [17,18]. Furthermore, age-adjusted neurocognitive development percentile cutoffs are suggested for a nuanced interpretation, assisting in the diagnosis of cognitive impairment by considering comorbidities, functionality, and biomarkers in the patient’s medical history. This aids in guiding potential treatments [17].

The cognitive domains impacted by SARS-CoV-2, including attention, visuospatial/executive functions, language repeat, and deferred recall, align with findings from other regional studies [19]. However, unlike some studies, we did not find that anxiety and depression influenced these outcomes [19]. Previous research indicates cognitive symptom recovery over periods exceeding 10 months, with executive function impairments affecting the return to work. In New York State, approximately 18% of long COVID patients were unable to resume work for more than a year [17,20]. By 2023, an estimated 4 million Americans with long COVID had not returned to work, incurring costs between 2.6 and 3.7 trillion dollars and exacerbating family income losses and health-related quality of life impacts [21].

Long COVID patients commonly exhibit fatigue (61.3%), insomnia symptoms (49.6%), and excessive daytime sleepiness (35.8%), with compromised sleep quality [22]. Cognitive function in roughly 70% of patients with sleep disorders, including insomnia, hypersomnia, and daytime sleepiness, may be affected [23]. The etiology is linked to neuroinflammation [4,22] and the potential involvement of immune mediators. Sleep deprivation exacerbates neuroinflammation by compromising the blood-brain barrier, facilitating the entry of viral antigens into the brain, and promoting inflammation [4,22]. However, it remains unclear whether inflammatory processes occur in brain regions associated with cognition and sleep, such as the prefrontal cortex or lateral hypothalamic area, post-COVID-19 diagnosis [22,23].

Sleep deprivation also heightens vulnerability to the virus by weakening the immune system [22]. The magnitude of sleep quality impairment seems consistent across different viral variants [22]. Sleep disorders are closely linked to the high prevalence of anxiety and depression [23]. The absence of significant sleep quality impairment in our study could be attributed to the primarily mild initial COVID-19 infection in our cohort, which did not necessitate hospitalization and was managed on an outpatient basis. In contrast, most studies reporting poor sleep quality involve hospitalized patients requiring intensive care and mechanical ventilation [22,23].

The prevalence of long COVID in the USA has shown minimal variation since January 2023 [20], although it has decreased compared to 2022. This decrease is attributed to a reduction in acute infection rates, the implementation of treatment measures such as antivirals, and improved vaccination coverage. Quality of life assessments in the UK depict a moderately severe functional decline, akin to advanced cancers, yet less severe than that experienced by stroke patients [20]. The risk of cognitive decline post-COVID-19 is 19 times higher, affecting 81% of patients in outpatient settings [20]. Due to inflammatory mechanisms and potential structural changes in the brain, COVID-19 may predispose individuals to neurodegenerative decline [24]. The risk of dementia post-COVID-19 is 2.33 times higher than that associated with influenza, based on data from 236,379 survivors [23]. These findings underscore the importance of sustained prevention measures [20]. In this context, neurorehabilitation, cognitive training, and ongoing psychological counseling are increasingly vital. Tele-rehabilitation has emerged as a viable alternative, offering support to patients at home and enhancing screening and case management [23,25].

The clinical manifestations of long COVID are diverse and multisystemic. Global healthcare providers face challenges in establishing effective treatments, and individuals with long COVID often experience stigmatization, resulting in both personal and societal consequences. These disparities can influence the disease trajectory. As a pathological condition, long COVID necessitates recognition and collective efforts in research and treatment by the scientific community and public health entities [21].

Limitations

This study’s context is specific to Latin America, particularly Chile, where socioeconomic and cultural differences may affect the results compared to other countries in the region. Additionally, the majority of the studied population had limited education, potentially influencing test performance. The small sample size restricts the conclusiveness of our findings, and the reliance on self-reported data introduces potential bias due to individual memory and perception. Furthermore, the study did not consider the potential effects of intervention strategies implemented during the research period on the observed outcomes.

## Conclusions

This study reveals a positive association between COVID-19 diagnoses at 11 months and cognitive and sleep impairments in outpatient individuals not requiring hospitalization. The infection predominantly affected cognitive domains such as attention, visuospatial/executive functions, language repetition, and deferred recall, without significantly impacting sleep quality. The underlying causes of these effects, potentially involving neuroinflammation in brain areas associated with sleep and cognition, warrant further investigation. Cognitive and sleep disturbances may hinder work performance and delay the return to a productive life, posing potential impacts on family economics and bio-psycho-social well-being that merit consideration in future research.
